# Impact of COVID-19 on the change in work conditions and career choices in general Vietnamese population

**DOI:** 10.3389/fpubh.2023.1106036

**Published:** 2023-04-14

**Authors:** Linh Phuong Doan, Linh Khanh Le, Vu Anh Trong Dam, Thuc Minh Thi Vu, Laurent Boyer, Pascal Auquier, Guillaume Fond, Bach Tran, Carl A. Latkin, Roger C. M. Ho, Cyrus S. H. Ho, Melvyn W. B. Zhang

**Affiliations:** ^1^Institute for Global Health Innovations, Duy Tan University, Da Nang, Vietnam; ^2^Faculty of Medicine, Duy Tan University, Da Nang, Vietnam; ^3^Institute of Health Economics and Technology, Hanoi, Vietnam; ^4^Research Centre on Health Services and Quality of Life, Aix Marseille University, Marseille, France; ^5^Bloomberg School of Public Health, Johns Hopkins University, Baltimore, MD, United States; ^6^Department of Psychological Medicine, Yong Loo Lin School of Medicine, National University of Singapore, Singapore, Singapore; ^7^Institute for Health Innovation and Technology (iHealthtech), National University of Singapore, Singapore, Singapore; ^8^Lee Kong Chian School of Medicine, Nanyang Technological University, Singapore, Singapore

**Keywords:** work condition, work satisfaction, work motivation, career choice, COVID-19, Vietnam

## Abstract

**Objectives:**

The onset of COVID-19 has resulted in both morbidity and mortality. It also has a consequential impact on the Vietnamese economy. Prior studies have examined the impact of COVID-19 on healthcare professionals’ career decisions. However, no study remains to have examined the work conditions and career choices in a general Vietnamese population. Our study aims to identify factors associated with the change in work conditions and career choices in general Vietnamese population.

**Methods:**

An online cross-sectional study between September 2021 through to November 2021 (during the Omicron COVID-19 pandemic). Snowball sampling method was utilized in recruiting the participants. The questionnaire used in this study included the following questions: (a) Socio-demographic information; (b) impact of COVID-19 on personal habits/daily expenses; (c) Current nature of work and impact of COVID-19 on work; (d) Impact of COVID-19 on career decisions. Data analysis was performed using STATA version 16. Descriptive analysis followed by Ordered logit regression was performed, to identify potential covariates.

**Results:**

Six hundred and fifty participants were recruited, of which only 645 completed the survey. The completion rate was 99.2%. This study demonstrated the impact that COVID-19 has on finances, as only 32% of those sampled reported that they were able to pay in full. 46.6% of the respondents have had a decrease in their overall household income. With regards to their employment and work characteristics, 41.0% reported a decrease in their work satisfaction and 39.0% reported having reduced motivation for work. Females were less likely to consider transiting from their current job to another field than male participants. Respondents who were married, had a higher level of commitment to their current job, and lower inclination to transition to another field. Respondents experiencing financial difficulties were more likely to consider a transition to another field/work.

**Conclusion:**

This is perhaps one of the first studies to have examined the impact of COVID-19 on work intentions regarding career choices and transitions in the general Vietnamese population. Future financial policies must take into consideration these factors.

## Introduction

The COVID-19 pandemic first affected Vietnam on the 23rd of January 2020 (1). The first wave of COVID-19 in Vietnam lasted till 16th April 2020, resulting in a total of 100 cases in the community (1). To date, Vietnam has undergone a total of 4 waves of COVID-19 infection. The first wave resulted from the spread of the original strain of the COVID-19 virus from Wuhan, China, and the 2nd wave was by the D614G variant, followed by the alpha and delta variants resulting in the 3rd and 4th waves. COVID-19 infections grew from 100 (first wave) to 601,349 by the end of the fourth wave (1). To date, as of 14th May 2022, there are a total of 517,648,631 cases globally with 6,261,708 deaths, according to the World Health Organization (2). Of these numbers, in Vietnam, there have been a total of 10,690,471 cases, with 43,063 deaths. In dealing with the recurrent waves of infection, the Vietnamese government has implemented several measures, including social distancing and the introduction of lockdowns, to curb the spread of the COVID-19 infection (1). The efforts undertaken by the Vietnamese government have been recognized to be highly effective, and have been widely discussed in the published literature, as well as in the international new media (3). The COVID-19 pandemic has, taken a toll on the employment of many individuals. According to the General Statistics Office of Vietnam (4), in 2021, the number of individuals aged 15 years old and above, who were employed was 49 million. There has been a decrease of 1 million individuals employed since the start of the pandemic (4). In 2021, there was also a decrease in the average monthly income of employees, that of 32 thousand Vietnamese Dong (4). Huong et al. (5), in their article that reviewed the employment situation in Vietnam highlighted that the onset of the COVID-19 pandemic in 2020, led to the highest unemployment rate within Vietnam in the last 10 years. They reported that female workers, those who were unskilled, migrant, and informal workers were the most affected by the pandemic (5). Workers in the following industries, hotels and food and beverages, were the most affected (5). The impact of unemployment not only affects the individual him/herself but their household, as in the early stages of the COVID-19 pandemic, it was reported that the overall income of many households was reduced by as much as 70% (5, 6). This has had a consequential impact on families with children. The impact on children is also heightened by the fact that there have been school closures during the different waves of infection. Given the pandemic’s impact on employment and the overall economy, the Vietnamese government needed to implement domestic stimulus and acquire funding from external organizations such as the World Bank and the International Monetary Fund (5). As of the first quarter of 2022, the General Statistics Office of Vietnam has reported a gradual recovery of the economy. The overall Gross Domestic Product (GDP) was expected to increase by 5.03% over the same period last year, but this growth rate was still lower than the 6.85% rate in the first quarter of 2019 (7). External factors, for example, the ongoing Russia-Ukraine conflict and the increasing price of natural commodities, were factors that might hamper overall economic growth (7). There was a reduction in the unemployment rate and the underemployment rates as the economy gradually improved (7).

COVID-19 has impacted the overall economy and the employment rates in Vietnam. Across the literature, studies have examined the impact of COVID-19 on individuals’ career choices. Jemini-Gashi et al. (8) have described how the current pandemic has affected young people. They sampled a total of 30 high school students, who were in the 12th grade, and reported that the participants reported themselves struggling with their career decision-making process in the times of the pandemic (8). A series of research has examined how the pandemic has affected career choices among healthcare professionals, or those embarking on a healthcare-related career. Wang et al. (9) examined the career intentions amongst a group of medical students from Hubei Providence in China. They reported that several factors mediated students’ decisions about their career choices. These factors include of their year/grade, their attitude toward healthcare and how the pandemic has affected their lives (9). Deng et al. (10), in their study which sampled a total of 1837 medical students, reported that 6.9% of those who were currently training to be a medical doctor, have had decreased willingness to be a doctor since the onset of the pandemic. They reported several variables that contributed to one’s willingness to continue their training as a doctor, and these include of younger age, lower household income, fewer depressive symptoms, those who were less exposed to negative pandemic information, and those who were more satisfied with their own major after the pandemic (10). Other studies such as Rajabimajd et al. (11), have in their qualitative review reported how the fear associated with COVID-19 was associated not only with increased anxiety about one’s future career, but also resulted in job insecurity, reduced job satisfaction, and increased turnover rates. Aside from these studies, there have been other studies that have explored one’s commitment and satisfaction to their job, and the associated factors. Chanana et al. (12), in their study that examined 181 private school teachers, reported that female teachers are had continuance commitment as compared to male teachers. Rožman et al. (13), who sampled a total of 785 employees in Slovenia companies, reported there to be gender differences in work satisfaction, work engagement and work efficiency. Other studies, such as that by Perades-Aguiree et al. (14) not only demonstrated that there were gender differences in levels of burnout, but also demonstrated that burnout and turnover intention to be factors affecting one’s job motivation. In Malik et al.’s (15) study, the authors also reported how factors like fear associated with COVID-19 infection, the long working hours and the lack of support at workplace affect one’s performance. Specific to the Vietnamese context, Thai et al. (16) have explored the job satisfaction and the associated factors among community healthcare workers during the COVID-19 pandemic. Of the 319 healthcare workers they sampled, they reported that one’s level of job satisfaction was low. The authors articulated the need for interventions addressing one’s level of job satisfaction. It is evident from these articles that the pandemic has had an impact on career choices. As aforementioned, it is thus evident that the COVID-19 pandemic has impacted employment and the overall economy. To date, apart from the previous article (1) that outlined the challenges faced by the Vietnamese workforce in the acute phase of the pandemic, there remains no other study that has examined the impact of COVID-19 on employment; and one’s decision to retain in the same job/organization or transition to another. Whilst there has already been research examining the changing attitudes toward career/career choices mainly amongst those training to become healthcare workers/doctors, there remains no study to date, that has examined how the career choices of the general population, and that in a low and middle income, like Vietnam has changed since the onset of the pandemic. It is thus the aim of this study to explore this. Prior studies have identified demographic factors like gender, and other factors relating to the fear of infection, burnout and turnover intention might affect one’s motivation, and it is crucial to conduct this current study to examine related factors. The findings revealed by this study would be of importance, as they would have resultant implications for businesses/organizations and might guide the formulation of policies.

## Methods

### Study design and sampling methods

To address the aims of the study, we conducted an online cross-sectional study between September 2021 to November 2021. This study was conducted when Vietnam was experiencing a resurgence of COVID-19 cases due to the spread of the Omicron variant. Snowball sampling method was utilized in recruiting the participants based on key initials across three regions: North, South, and Middle, including large metropolitans such as Ha Noi capital city and Ho Chi Minh city.

The formula for estimating a population proportion was used for the sample size for this study. In particular, the expected proportion of people who have high organizational commitments was 0.72 [according to a previous study in Iran (17)], the confidence level was 0.95, and the relative precision was 0.05. Thus, leading to the necessary sample size was 598 respondents. Furthermore, to avoid participants the incomplete questionnaire or dropout during the research, 10% of the sample size was added, thus resulting in the data from 658 participants needing to collect. Finally, at the end of the participant recruitment, data from 650 participants were collected, of which only 645 completed the survey (with the completion rate was 99.2%).

In this study, the research instrument was developed based on a standard procedure. Firstly, we conducted a systematic review to find out the gap as well as the important aspects that have been indicated from the previous research and then developed frames of the questionnaire for the study. Furthermore, several experts in the COVID-19 field were invited to jointly discuss the translation, language, and logical order of the questionnaire. Before collecting the data process, the questionnaire was designed and piloted by 15 staff members from the Vietnam Young Physician Association to test any text, and any technical issue one more time. Finally, the questionnaire after the revision was sent to a group of 20 participants from the community/general population with various occupations such as students, white-collar workers, and freelancers. After completing the questionnaire, these participants were told to share the linked questionnaire with their colleagues and acquaintances. Participants took 20–30 min for the completion of the questionnaire study.

Informed consent was acquired from all participants. The benefits and risks associated with their participation in the study were informed to all. Due to the voluntary nature of the study, participants were told that they could leave/ withdraw at any point of time. A web-version questionnaire database, that of Survey Monkey, was used to disseminate the questionnaire and track the data.

### Inclusion and exclusion criteria

Participants were eligible to participate in the study if they were (a) aged 16 years and above, (b) able to provide informed consent, (c) lived in Vietnam at least 6 months (d) were able to access the survey link using a computer/mobile device.

### Measurements

The questionnaire used in this study included the following questions: (a) Socio-demographic information; (b) impact of COVID-19 on personal habits/daily expenses; (c) Current nature of work and impact of COVID-19 on work; (d) Impact of COVID-19 on career decisions. Some of these questionnaires were included in consideration of some of the variables aforementioned in the introductory paragraph that affected one’s decision to remain or make a transition in one’s job.

In the following section on Outcome Variables, further details on each questionnaire are provided.

### Outcome variables

#### Occupational intention (impact of COVID-19 on career decisions)

A questionnaire comprising five items was designed to ascertain the participants’ occupational intentions:

1. Are you determined to complete your job at your current workplace and stick to this job?
2. Have you considered transferring to another unit/division within the same organization?
3. Are you considering switching to another organization (that deals with the same nature of business)?
4. Have you considered switching to another area of work, but remaining in the same line of business?
5. Have you considered moving on to another new job (in a different field)?

Participants were told to rate how each of the statements. Commitment to the current job is ascertained based on their responses to question 1 and 5.

### Covariate

#### Socioeconomic status

Respondents reported their socio-demographic information questions, including age, gender (male/female), marital status (single, others), living location (urban areas, town, rural/mountainous areas), main income/month (under 5 million VND, 5–10 million VND, and 10 million VND and above), and monthly household income *per capita* (under 5 million VND, 5–10 million VND, and 10 million VND and above).

#### Impact of COVID-19 on personal demand

Seven questions were included to ascertain the impact of COVID-19 on personal habits and expenses. These questions focused on the following areas: eating, electricity/water/bills, education, transport, clothes, health care, and family expenses. Participants were told to rate their responses as to whether they have had (a) enough to pay in full; (b) sufficient to pay partially; (C) or totally had not enough to finance.

#### Impact of COVID-19 on work

Variables that assessed the impact of COVID-19 on employment included having a cost-of-living allowance (yes/no); Changes in income and spending (wage, allowance, bonus, other incomes at work, other sources of incomes outside the workplace, monthly income of the whole family), average working time per day (less than 8 h, 8–10 h, and more than 10 h), average workload (no changed or increased), change in work satisfaction level (no change, decreased, increased), and change in work motivation level (no change, decreased, increased).

To ascertain the impact of COVID-19 on one’s current work, based on the previous study by Tran et al. (18) 10 questions were developed, including:

1. Daily work intensity.
2. Level of work-related stress and fatigue.
3. Health risks caused by work.
4. The community’s stigma with the work I’m doing.
5. Your ability to endure and cope with external work pressures.
6. Process and professionalism of routine work.
7. Complexity in coordination between colleagues, and between departments.
8. New knowledge and skills for work.
9. Ability to complete assigned tasks.
10. Ability to ensure safe means of work.

All above 10 statements were rated using a 5-point Likert scale (1 = Completely unchanged; 2 = Changed little; 3 = Changed relatively much; 4 = Changed a lot; 5 = Changed extremely, beyond processing capacity).

### Data analysis

We analyzed the data using STATA version 16 (Stata Corp. LP, College Station, United States). To deal with the issues of missing data, we used the Listwise Deletion method to clean data before analyzing it (19). Continuous variables were presented as mean and standard deviation (SD), while categorical variables were presented as frequencies with percentages.

Potential covariates for full models “commitment to the current job” and “Consider moving job to another field” included “individual characteristics, the impact of COVID-19 on personal demand, the impact of COVID-19 on work, and working condition characteristics.” We used multivariate Ordered logit regression to confirm factors associated with “commitment to the current job.” These models were then combined with the stepwise forward strategies to produce reduced models with *p* < 0.2 as the threshold for included variables (20). The value of p (P) < 0.05 was considered statistically significant.

## Results

Six hundred and fifty participants were recruited, of which only 645 completed the survey. The completion rate was 99.2%. Table 1 summarized the characteristics of the sampled individuals and the impact of COVID-19 on personal finances. 67.3% of the participants were aged between 16 and 20 years old, with 67.7% female respondents. Of those sampled, 9.5% were married, 55.3% resided in the urban area, 28.4% resided in rural/mountainous/island areas, 82.5% had tertiary education and most of the participants were students (53.2%) 85.1% of the participants had a monthly income per month which was under 5 million VND (Vietnamese Dong). Most of those sampled (85.1%) had a monthly household income *per capita* under 10 million VND, and 29.2% of those sampled had an income of 10 million VND or above.

**Table 1:** Characteristics of recruited participants and impact of COVID-19 on personal finances.

Characteristics		*n*	%
Age group	16–20 years old	435	67.3
	21–25 years old	137	21.2
	More than years old	74	11.5
Gender	Male	208	32.3
	Female	436	67.7
Marital status	Single/Divorced/Widowed	584	90.5
	Married	61	9.5
Education	Tertiary	532	82.5
	Intermediate/College	66	10.2
	Post graduate	18	2.3
	Other	29	4.5
Occupation	Student	346	53.2
	White-collar worker	155	23.9
	Freelancer	149	22.9
Living location	Urban areas	359	55.3
	Town	106	16.3
	Rural/Mountainous/Island areas	184	28.4
Main income/month	Under 5 million VND	532	85.1
	5–10 million VND	62	9.9
	10 million VND or above	31	5.0
Monthly household income *per capita*	Under 5 million VND	322	49.9
	5–10 million VND	188	29.2
	10 million VND or above	135	20.9
Personal demand during COVID-19			
Eating	No changed	300	46.2
	Decreased	262	40.3
	Increased	88	13.5
Electricity, water, bills	No changed	246	37.9
	Decreased	84	12.9
	Increased	320	49.2
Education	No changed	367	56.5
	Decreased	207	31.9
	Increased	76	11.7
Transport	No changed	204	31.4
	Decreased	411	63.2
	Increased	35	5.4
Clothes	No changed	348	53.5
	Decreased	274	42.2
	Increased	28	4.3
Health care	No changed	355	54.6
	Decreased	142	21.9
	Increased	153	23.5
Family expenses	No changed	203	31.2
	Decreased	254	39.1
	Increased	193	29.7
Affordability to pay household expenses	Enough to pay in full	208	32.0
	Sufficient to pay part	270	41.5
	Totally not enough	172	26.5

Regarding personal finances, most participants reported that their finances for food, education, and healthcare did not change during COVID-19. 49.2% of the participants had to spend more on electricity/water/bills. Pertaining to individual’s affordability, only 32% of the respondents had the ability to pay in full.

Table 2 provides an overview of the impact of COVID-19 on individuals’ income, their workload, and work-related satisfaction/motivation. 11.6% of the participants reported that they have received an allowance. Regarding individuals’ income, most participants reported no changes in their wages, allowances, bonus, or other income sources at or outside the workplace. However, 46.6% of the participants reported a decrease in the monthly income of the whole family. In terms of workload, 75.1% of the participants reported working on average 8 h or less. 81.3% reported no change in their average workload. 41.0% reported a decrease in their work satisfaction and 39.9% reported reduced motivation for their work. Participants reported there being no changes or little changes in their work.

**Table 2:** Impact of COVID-19 on income, workload, and work-related satisfaction and motivation.

Characteristics		*n*	%
Having cost-of-living allowance		75	11.6
Changing in income and spending			
Wage	No changed/Increased	418	65.1
	Decreased	224	34.9
Allowance	No changed/Increased	517	80.8
	Decreased	123	19.2
Bonus	No changed/Increased	510	80.6
	Decreased	123	19.4
Other incomes at work	No changed/Increased	506	79.6
	Decreased	130	20.4
Other incomes outside the workplace	No changed/Increased	489	77.4
	Decreased	143	22.6
Monthly income of the whole family	No changed/Increased	342	53.4
	Decreased	298	46.6
Average working time per day	8 h or less	482	75.1
	8–10 h	118	18.4
	More than 10 h	42	6.5
Average workload	No changed	526	81.3
	Increased	121	18.7
Work satisfaction level	No changed	356	55.1
	Decreased	265	41.0
	Increased	25	3.9
Work motivation level	No changed	321	49.6
	Decreased	258	39.9
	Increased	68	10.5
		Mean	SD
Recent changes in work (range: 1–5)	Daily work intensity	2.0	0.9
	Level of work-related stress and fatigue	2.0	0.9
	Health risks caused by work	1.9	0.9
	The community’s stigma with the work I’m doing	1.4	0.7
	Your ability to endure and cope with external work pressures	1.8	0.9
	Process and professionalism of routine work	1.7	0.9
	Complexity in coordination between colleagues, and between departments	1.7	0.9
	New knowledge and skills for work	1.8	0.9
	Ability to complete assigned tasks	1.7	0.9
	Ability to ensure safe means of work	1.7	0.8

**Figure 1:**
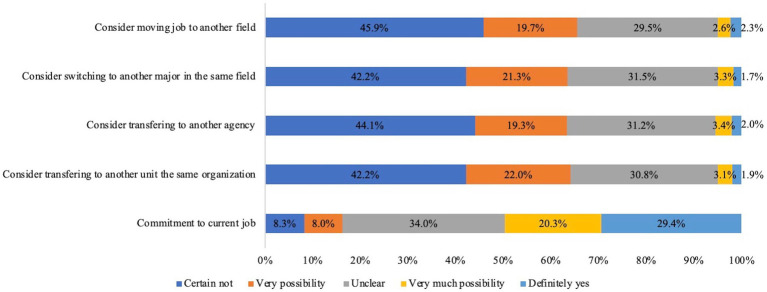
Overview of intentions to change career.

Figure 1 provides an overview of participants’ intentions in changing their careers. It is apparent that most of the participants intend to keep their existing job, with few indicating an intention to change their jobs.

Table 3 presented the odds ratio (OR) and 95% Confident Intervals (CI) from the Ordered logistic regression analysis. The main outcomes from the regression analysis, which were statistically significant were that female participants were less likely to consider moving jobs to another field than male participants (OR = 0.69; 95%CI = 0.48; 0.98); respondents who were married had a higher level of commitment to the current job (OR = 3.09; 95%CI = 1.67; 5.73) and lower level of considering the moving job to another field (OR = 0.48; 95%CI = 0.25; 0.93). The freelancer was likely to consider moving a job to another field (OR = 1.77; 95%CI = 1.18; 2.65). Increasing health care demand was the factor that was likely to consider moving jobs to another field (OR = 1.53; 95%CI = 1.02; 2.30).

**Table 3:** Factor associated with career choices of participants.

Factors	Commitment to current job		Consider moving job to another field	
	From 1 “Certainly not” to 5 “Definitely yes”		From 1 “Certainly not” to 5 “Definitely yes”	
	OR	95%CI	OR	95%CI
Socio-economic
Gender (Female vs. Male-Ref)			0.69**	0.48; 0.98
Marital status (Married vs. Single/Divorced / Widowed-Ref)	3.09***	1.67; 5.73	0.48**	0.25; 0.93
Education (vs. Tertiar -Ref)				
Intermediate/College	0.85	0.47; 1.54	1.56	0.85; 2.87
Post graduate	1.02	0.36; 2.92	1.23	0.44; 3.44
Other	0.59	0.28; 1.22	1.41	0.64; 3.08
Location (vs. Urban areas -Ref)				
Town			0.70	0.43; 1.13
Rural/Mountainous/Island areas			1.16	0.79; 1.69
Occupation (vs. Students -Ref)				
White-collar worker	0.78	0.53; 1.15	0.86	0.56; 1.31
Freelancer	0.75	0.51; 1.10	1.77***	1.18; 2.65
Personal demand during COVID-19				
Health care (vs. No changed -Ref)				
Decreased			1.03	0.66; 1.59
Increased			1.53**	1.02; 2.30
Affordability to pay household expenses (vs. Enough to pay in full -Ref)				
Sufficient to pay part	0.74	0.51; 1.08	2.21***	1.48; 3.30
Totally not enough	0.70*	0.46; 1.05	1.96***	1.26; 3.05
Working conditions
Having cost-of-living allowance (Yes vs. No-Ref)	1.40	0.85; 2.28		
Changes in income and spending (vs. Decreased vs. No changed/Increased-Ref)				
Wage	0.51***	0.33; 0.79		
Bonus			1.48	0.91; 2.38
Other incomes outside the workplace	0.59**	0.37; 0.92		
Monthly income of the whole family	1.69**	1.12; 2.54	0.67**	0.45; 1.00
Recent changes in work (unit: score)				
Daily work intensity	1.47***	1.16; 1.86		
Level of work-related stress and fatigue	0.80*	0.64; 1.00		
Health risks caused by work			0.82	0.64; 1.05
The community’s stigma with the work I’m doing	0.74**	0.59; 0.94	1.52***	1.14; 2.02
Ability to ensure safe means of work			1.43***	1.12; 1.83
Work satisfaction level (vs. No changed-Ref)				
Decreased			1.67***	1.17; 2.38
Increased			1.33	0.55; 3.19
Work motivation level (vs. No changed-Ref)				
Decreased	0.59***	0.42; 0.84		
Increased	1.28	0.75; 2.18		

***p < 0.01, **p < 0.05, *p < 0.1.

Participants who were able to partially pay (OR = 2.21; 95%CI = 1.48; 3.30) or have had totally not enough finances to pay household expenses (OR = 1.96; 95%CI = 1.26; 3.05) were more likely to consider moving a job to another field than those who had enough to pay in full. A reduction in the overall monthly income of the whole family also had the opposite impact, in that it affected the commitment to their current job (OR = 1.69; 95%CI = 1.12; 2.54) and increased their consideration of moving job to another field (OR = 0.67; 95%CI = 0.45; 1.00). Meanwhile, decreases in wages (OR = 0.51; 95%CI = 0.33 l 0.79) and other incomes outside the workplace (OR = 0.59; 95%CI = 0.37; 0.92) were likely to decrease the commitment to the current job. Changing in daily work intensity was likely to increase the commitment to the current job (OR = 1.47; 95%CI = 1.16; 1.86), by contrast, change in the community’s stigma with the work I’m doing and ability to ensure safe means of work had the opposite impact. Decreasing work satisfaction level also resulted in individuals’ consideration in moving job to another field (OR = 1.67; 95%CI = 1.17; 2.38), meanwhile, decreasing work motivation level had a negative effect on commitment to current job (OR = 0.59; 95%CI = 0.42; 0.84).

## Discussion

This study is perhaps one of the first to examine the impact of COVID-19 on employment in Vietnam, and how the pandemic has affected individuals’ career choices. There are several key findings arising from this study. First, this study demonstrated the impact that COVID-19 has on finances, as only 32% of those sampled reported that they were able to pay in full. 46.6% of the respondents have had a decrease in their overall household income. Regarding their employment and work characteristics, 41.0% reported a decrease in their work satisfaction and 39.0% reported having reduced motivation for work. We found that females were less likely to consider transiting from their current job to another field than male participants (OR = 0.69; 95%CI = 0.48; 0.98). Respondents who were married had a higher level of commitment to their current job, and a lower inclination to transition to another field. Respondents experiencing financial difficulties were more likely to consider a transition to another field/work. Individuals who were free-lancing were more likely to make a career switch (OR = 1.77; 95%CI = 1.18; 2.65). More importantly, if there was an increase in healthcare demands in a job, individuals were more likely to switch jobs. In addition, changes in work intensity (OR = 1.47; 95%CI = 1.16; 1.86) and reduced work satisfaction also resulted in individuals transitioning to another job.

Huong et al. (5) in their article have provided an overview of the economic and employment issues that Vietnam was afflicted with, 6 months after the start of the pandemic. In their article, they highlighted some of the measures that the Vietnamese government undertook, and these measures were classified into general solutions, urgent and long-term solutions (5). The general solutions included expanding the domestic market, developing an attractive business market, and identifying opportunities and challenges to help stimulate economic growth (5). Urgent solutions included that of granting extensions for payment of taxes and other duties, financial assistance that allows lending institutions to lower borrowing rates, and debt restructuring (5). At the time of conduct of this study, these “urgent” measures ought to have been in place, but a good proportion of the respondents sampled still report having difficulties in their personal finances, and their ability to pay in full for their financial obligations. We postulate that whilst tremendous efforts have been undertaken by the Vietnamese government, the relief measures might not be adequate given the disruptions brought upon by the repeated waves of COVID-19 infections. Despite these challenges, Huong et al. (5) have also highlighted the long-term measures that the Vietnamese government has strategize, such as recognizing the importance of developing new policies and legislation to facilitate new business models, to restructure the economy, and to constantly innovate using science and technologies. It remains of importance for there to be further evaluations following the implementation of these policies, to see if they have any impact on one’s decision to continue at their same job or make a transition to another job.

One of the key findings was a decrease in motivation and confidence to work, and certain factors (such as gender, marital status, and financial stability) were associated with whether one has intentions to transition within the same job environment, or onto another job. Previous studies have highlighted the impact that COVID-19 has on those working in healthcare. The onset of the COVID-19 pandemic has resulted in longer working hours for healthcare workers. Healthcare workers also needed to constantly adapt to new demands at their job in view of the new disease variants/updated knowledge about the virus (21). The toll on healthcare workers was tremendous in that they also needed to deal with the increased mortality and had to dedicate time to their own families while at the frontline dealing with the pandemic (21). Tran et al. (18) have proposed a model that demonstrated how the interaction of different factors would increase healthcare workers’ toll. Such factors include individual predisposing factors (like health status, family attachment, security), psychosocial outcomes of healthcare jobs, and the working environment. In a similar vein, the findings from the current study can be conceptualized in much the same way. As the pandemic progresses, the challenges that healthcare workers and workers in other occupation experience might differ. Future research should examine the impact of the protracted pandemic on workers, with repeated assessment of their intentions to stay on or leave their jobs. This would guide the implementation of relevant policies to retain or alleviate the pressures on healthcare workers.

One of the key findings arising from this study was the identification of individual factors that predispose whether one would make a job transition. Some of the factors that we have identified included those of gender, marital status, financial security, work intensity and demands and one’s level of satisfaction with their work. With regards to gender differences, Rožman et al. (13) reported there to be gender differences in terms of work satisfaction, engagement, and efficiency when they sampled a total of 785 employees. However, in contrast to our findings, their study found females to have lower satisfaction during the pandemic, as they had to meet not only their job demands, but handle higher household demands, and provide care for their family. Similarly, according to UN Women (22), they stated that the pandemic has a huge impact for females, as compared to males, given that females are likely to face difficulties relating to having unpaid care, violence, overload of work and job loss. Our current findings are contrary to these findings and are unique and suggest that future research is needed, to explore this difference, as it is to be expected that Vietnamese female workers would also need to handle more responsibilities, but they remain less likely than males to be inclined in making a job transition. It is of important to study and examine organizational policies that help maintain their levels of satisfaction at the workplace. Pertaining to financial security, work intensity and work demands, our findings are in-line with the findings of prior studies. The provision of a good salary package has been shown to be a strong factor that affects the organizational committee of an employee (23). Regarding work demands, studies done among healthcare workers have shown how increased work demands not only leads to a deterioration in physical well-being, but could have consequential impact on one’s mental health, leading to employees developing depression and anxiety (15). Thus, these would have an impact on one’s work quality and job satisfaction. Factors relating to the working environment that might predispose individuals to consider a change of their job include one’s satisfaction and confidence in the job. The demographic variables we identified highlighted that there ought to be more targeted interventions focusing on groups that are more vulnerable. Workplace-based interventions should be considered, to better help employees to adjust to their workplace, and to avoid decisions to leave. Bienkowska et al. (24) highlighted how different human resource strategies could help in ensuring employee’s well-being and strengthen organizational performance. Some of these strategies include, enabling hybrid modality of work and helping employees feel safe about their health.

There are several strengths of this study. This study is perhaps the first study to have examined the impact of COVID-19 on work conditions and decisions to transition between careers among a population sampled from all over Vietnam. The sample size was sizeable, and the completion rate was adequate. Statistical analysis also managed to identify factors that might increase the odds of one making a career transition. Despite these strengths, there remain several limitations. The age of the overall sample was relatively young, 16–20 years old. It will be important to sample a wider range of ages, and individuals from different jobs/organizations. The nature of the sampling method might affect the overall representativeness of the sample. Most of the information that we have obtained was based on self-report, and hence there might be recall biases. It would be beneficial to track these participants over a period of time and determine if there are any changes in their responses, as the COVID-19 pandemic develops.

## Conclusion

In conclusion, this study has shed light on the impact of COVID-19 on employment and career choices among individuals in Vietnam. The findings suggested that the pandemic has had a significant effect on household finances, work satisfaction, and motivation. Factors such as gender, marital status, and financial stability were found to be associated with one’s intentions to transition within or outside of their current job environment. The study also highlighted the need for targeted interventions to support vulnerable groups and workplace-based strategies to promote employee well-being and organizational performance. While this study has several strengths, such as a sizeable sample size and adequate response rate, there are also limitations, including a relatively young age range and potential recall biases in self-reported data. Future research should aim to address these limitations and examine the impact of the protracted pandemic on healthcare workers and other vulnerable populations. Overall, this study contributes to a better understanding of the impact of COVID-19 on employment and career choices in Vietnam and provides insights that may inform policy interventions and organizational strategies to support individuals and communities affected by the pandemic.

## Data availability statement

The raw data supporting the conclusions of this article will be made available by the authors, without undue reservation.

## Ethics statement

The studies involving human participants were reviewed and approved by the study was approved by the scientific committee of the Youth Research Institute (Code: ĐT.KXĐTN 22–11). Participation was completely voluntary. Collected data were saved in a secured system and only served the study purposes. Written informed consent to participate in this study was provided by the participants’ legal guardian/next of kin.

## Author contributions

LD: conceptualization, resources, supervision, writing – review and editing. LL: writing – original draft, writing – review and editing. VD: data curation, formal analysis, and resources. TV: writing – original draft, writing – review and editing. LB: writing – original draft and formal analysis. PA: writing – original draft and formal analysis. GF: writing – original draft, writing – review and editing. BT: conceptualization, resources, supervision, writing – review and editing. CL: conceptualization, resources, and supervision. RH: writing – original draft, writing – review and editing. CH: writing – original draft, writing – review and editing. MZ: writing – original draft, writing – review and editing. All authors contributed to the article and approved the submitted version.

## Funding

This study was supported by Actionaid Vietnam, Vingroup Innovation Foundation (VINIF) (Grant No. VINIF.2020.COVID-19.DA03), NUS Department of Psychological Medicine (R-177-000-100-001/R-177-000-003-001/ R177000702733) and NUS iHeathtech Other Operating Expenses (R-722-000-004-731).

## Conflict of interest

The authors declare that the research was conducted in the absence of any commercial or financial relationships that could be construed as a potential conflict of interest.

## Publisher’s note

All claims expressed in this article are solely those of the authors and do not necessarily represent those of their affiliated organizations, or those of the publisher, the editors and the reviewers. Any product that may be evaluated in this article, or claim that may be made by its manufacturer, is not guaranteed or endorsed by the publisher.
